# Isolated unilateral absence of the pulmonary artery

**DOI:** 10.1002/ccr3.2515

**Published:** 2019-10-29

**Authors:** Kenji Kanno, Takamitsu Maehara, Taketsugu Yamamoto, Daiji Nemoto, Hironori Kigoshi, Munetaka Masuda

**Affiliations:** ^1^ Department of General Thoracic Surgery Yokohama‐Rosai Hospital Yokohama‐shi Kanagawa Japan; ^2^ Department of Surgery Yokohama City University Yokohama‐shi Kanagawa Japan

**Keywords:** hemoptysis, unilateral absence of the pulmonary artery, vascular abnormality

## Abstract

Isolated unilateral absence of the pulmonary artery (UAPA) is a rare malformation. It is associated with respiratory symptoms, such as dyspnea or hemoptysis. We suggest that surgical treatment should be positively considered in patients with UAPA who are severely symptomatic and who have no other cardiovascular or respiratory comorbidities.

## Graphical abstract

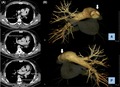

A 41‐year‐old Japanese man was referred to us because of hemoptysis. Chest roentgenogram showed a mediastinal shift with no consolidations (Figure 1). Chest‐enhanced computed tomography showed the absence of the left pulmonary artery from its origin, but the presence of a multitude of small vessels at the hilum from the bronchial, left internal thoracic, and left inferior phrenic arteries (Figure 2). No other cardiovascular malformations were detected. The diagnosis was isolated unilateral absence of a pulmonary artery (UAPA), which we considered to be the cause of the hemoptysis. He underwent left pneumonectomy, as there was no main pulmonary artery. In contrast, pulmonary venous drainage was anatomically normal. He had no recurrent symptoms during 21 months of follow‐up.

**Figure 1:**
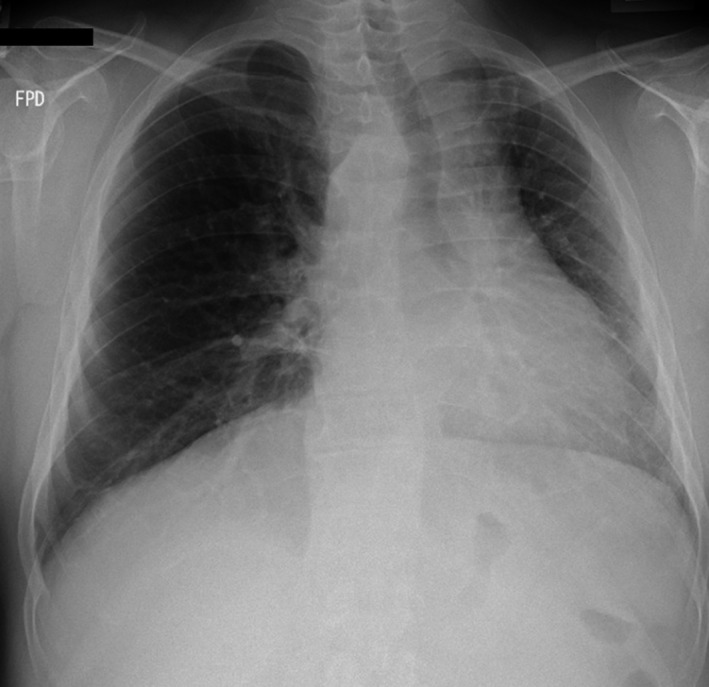
Preoperative images. Chest roentgenogram showed a mediastinal shift

**Figure 2:**
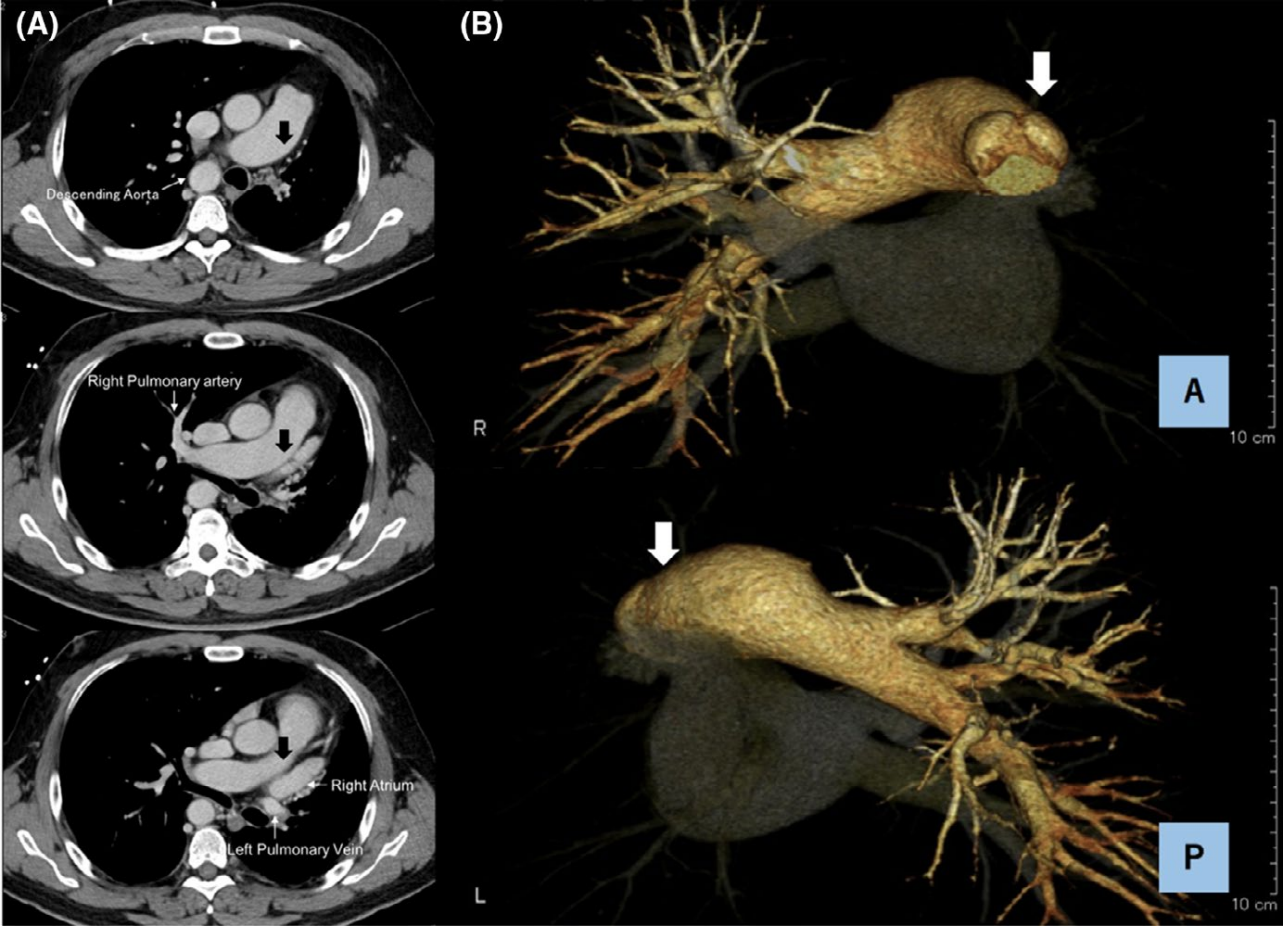
Preoperative images. A, Contrast‐enhanced computed tomography (CT) shows the absence of the left pulmonary artery (black arrows). B, Three‐dimensional CT clearly shows the absence of the left pulmonary artery from its origin in an anterior view and posterior views (white arrows)

Unilateral absence of the pulmonary artery is a rare anomaly and usually occurs in association with other cardiovascular malformations. It is associated with respiratory symptoms, such as dyspnea or hemoptysis; however, approximately 30% of patients with UAPA remain asymptomatic.^1^ One study reported successful selective embolization for UAPA with hemoptysis.^2^ Besides that, surgical treatments, such as lobectomy or pneumonectomy, are considered for patients with UAPA who are severely symptomatic. Our patient has been free of hemoptysis after pneumonectomy and experiences no dyspnea even with exercise.

## CONFLICT OF INTEREST

None declared.

## AUTHOR CONTRIBUTIONS

Kenji Kanno wrote the initial draft of the manuscript. Takamitsu Maehara supported writing the manuscript. Taketsugu Yamamoto supported writing the manuscript. Daiji Nemoto assisted in the preparation of the manuscript. Hironori Kigoshi assisted in the preparation of the manuscript. Munetaka Masuda is a supervisor and edited the manuscript. All authors reviewed and approved the final manuscript.

## INFORMED CONSENT

Informed consent has been obtained from the patient.
